# Psychosocial factors associated with the risk of developing psychosis in a Mexican general population sample

**DOI:** 10.3389/fpsyt.2023.1095222

**Published:** 2023-02-16

**Authors:** Tecelli Domínguez-Martínez, Tamara Sheinbaum, Ana Fresán, Lourdes Nieto, Steven R. López, Rebeca Robles, Ma del Carmen Lara, Camilo de la Fuente-Sandoval, Neus Barrantes-Vidal, Ricardo Saracco, Karina Franco-Paredes, Felipe Díaz-Reséndiz, Mauricio Rosel

**Affiliations:** ^1^Centro de Investigación en Salud Mental Global, Instituto Nacional de Psiquiatría “Ramón de la Fuente Muñiz”-UNAM, Mexico City, Mexico; ^2^Dirección de Investigaciones Epidemiológicas y Psicosociales, Instituto Nacional de Psiquiatría “Ramón de la Fuente Muñiz”, Mexico City, Mexico; ^3^Subdirección de Investigaciones Clínicas, Instituto Nacional de Psiquiatría “Ramón de la Fuente Muñiz”, Mexico City, Mexico; ^4^Department of Psychology, University of Southern California, Los Angeles, CA, United States; ^5^Facultad de Medicina, Benemérita Universidad Autónoma de Puebla, Puebla, Mexico; ^6^Laboratorio de Psiquiatría Experimental, Instituto Nacional de Neurología y Neurocirugía, Mexico City, Mexico; ^7^Departament de Psicologia Clínica i de la Salut, Universitat Autònoma de Barcelona, Barcelona, Spain; ^8^Sant Pere Claver - Fundació Sanitària, Barcelona, Spain; ^9^Centre for Biomedical Research Network on Mental Health (CIBERSAM), Instituto de Salud Carlos III, Barcelona, Spain; ^10^Universidad de Guadalajara-Centro Universitario del Sur, Guadalajara, Mexico; ^11^Clínica de Esquizofrenia, Instituto Nacional de Psiquiatría “Ramón de la Fuente Muñiz”, Mexico City, Mexico

**Keywords:** clinical high-risk, prodrome, psychosis, family functioning, cannabis use, life adversities, childhood trauma and adversity, early detection and prevention

## Abstract

Epidemiological evidence has linked an array of sociodemographic and psychosocial factors with an increased risk of developing psychosis. However, research in samples from low- and middle-income countries is still scarce. This study used a Mexican sample to explore (i) sociodemographic and psychosocial differences between individuals with and without a positive screen for Clinical High-Risk for psychosis (CHR), and (ii) sociodemographic and psychosocial factors associated with screening positive for CHR. The sample consisted of 822 individuals from the general population who completed an online survey. Of the participants, 17.3% (*n* = 142) met the CHR screening criteria. Comparisons between those who screened positive (CHR-positive group) and those who did not (Non-CHR group) showed that participants in the CHR-positive group were younger, had a lower educational level, and reported more mental health problems than the Non-CHR group. Furthermore, relative to the Non-CHR group, the CHR-positive group had a greater prevalence of medium/high risk associated with cannabis use, a higher prevalence of adverse experiences (bullying, intimate partner violence, and experiencing a violent or unexpected death of a relative or friend), as well as higher levels of childhood maltreatment, poorer family functioning, and more distress associated with the COVID-19 pandemic. Groups did not differ in sex, marital/relationship status, occupation, and socio-economic status. Finally, when examined in multivariate analyses, the variables associated with screening positive for CHR were: having an unhealthy family functioning (OR = 2.75, 95%CI 1.69–4.46), a higher risk associated with cannabis use (OR = 2.75, 95%CI 1.63–4.64), a lower level of education (OR = 1.55, 95%CI 1.003–2.54), having experienced a major natural disaster (OR = 1.94, 95%CI 1.18–3.16), having experienced a violent or unexpected death of a relative or friend (OR = 1.85, 95%CI 1.22–2.81), higher levels of childhood emotional abuse (OR = 1.88, 95%CI 1.09–3.25), physical neglect (OR = 1.68, 95%CI 1.08–2.61), and physical abuse (OR = 1.66, 95%CI 1.05–2.61), and higher COVID-related distress (OR = 1.10, 95%CI 1.01–1.20). An older age was a protective factor for screening positive for CHR (OR = 0.96, 95%CI 0.92–0.99). Overall, the findings highlight the importance of examining potential psychosocial contributors to psychosis vulnerability across different sociocultural contexts to delineate risk and protective processes relevant to specific populations and better target preventive intervention efforts.

## 1. Introduction

Psychotic spectrum disorders are complex syndromes characterized by a disconnection from reality and the presence of varied symptoms such as delusions, hallucinations, distortion of the thought process, disorganized thinking and/or motor behavior, negative symptoms (i.e., affective blunting, alogia, asociality, anhedonia, and avolition), cognitive deficits, and functional impairment (1, 2). These disorders commonly appear during the transition period between adolescence and adulthood and cause impairments across multiple life domains, generating significant personal, social, health, and economic costs (3).

Research has demonstrated that psychotic-like experiences are common in the general population, and psychotic symptoms are not exclusive to psychotic disorders but are transdiagnostic and may be present in different mental disorders (4). Studies on the prevalence of psychotic-like experiences (i.e., milder forms of positive psychotic symptoms in the absence of a psychotic disorder) have produced mixed findings that vary according to place and ethnicity (5). These studies have generally found a prevalence of between 5 and 10% in the general population (6, 7) and in adolescents (8). Furthermore, recent studies on the impact of the COVID-19 pandemic and lockdown have reported an increased prevalence of psychotic-like experiences in adolescent and general population samples (9–11). Although psychotic-like experiences tend to be transitory in around 80% of those who experience them, approximately 20% develop persistent psychotic-like experiences, and 7% a psychotic disorder (4, 5).

The onset of psychosis is usually preceded by premorbid developmental alterations and can start with a prodromal phase characterized at first by non-specific symptoms, and then by subthreshold positive, negative, affective, and behavioral symptoms (12). Over the last decades, increasing research has focused on the early detection and intervention in psychosis paradigm, based on the possibility of identifying adolescents and young adults at the preclinical or subclinical phase, before the onset of the first episode of psychosis. These individuals are considered to be at imminent risk for developing psychotic disorders, also known as “Clinical High Risk” (CHR), “Ultra-High Risk” (UHR), or At-Risk Mental States for Psychosis (13, 14). The CHR stage of psychosis is typically defined by the presence of either attenuated (subclinical) or transient positive psychotic symptoms (i.e., lasting no more than a week, with spontaneous full recovery), along with a significant decrease in psychosocial functioning in the last year (15–17). A recent systematic review and meta-analysis indicated a prevalence of 1.7% of CHR states in the general population of adolescents and young adults, and 19.2% in those entering mental health services for the first time (18). Although it has been suggested that the prevalence of CHR may vary according to gender (19), age (20), and geographical location (21), the impact of these factors has not been clearly established (18).

Psychotic disorders are considered polygenic and multifactorial (12), likely resulting from the synergistic co-participation of genetic and environmental factors (22). Although the genetic aspects of these disorders have received considerable attention (23), there is growing interest in the contribution of sociodemographic and psychosocial factors to the etiology and course of psychosis (24–26). To date, epidemiological evidence has linked an array of such factors with an increased risk for psychosis. These include male gender, age between 15 and 35 years, migrant or ethnic minority status, urbanicity, difficult socioeconomic conditions or household poverty, and cannabis use (12, 19, 27–33). In addition, strong evidence demonstrates that a range of childhood adversities and traumatic life events are pluripotent risk factors associated with the development of psychosis in adolescence and adulthood (34–42). The most prominent adversity experiences associated with psychosis risk include parental separation or loss (2, 12, 41, 43–45), child maltreatment (sexual, physical, and emotional abuse, and physical and emotional neglect), and peer victimization or bullying (41, 46–52). Overall, the evidence appears to be especially robust for interpersonal forms of adversity, whereas that for non-interpersonal traumatic events (such as accidents or natural disasters) is more limited and inconsistent (53).

The family environment is another important factor in psychosocial research on psychosis (54). Although characteristics of the family environment have generally received more attention as predictors of relapse (55, 56), there is also evidence suggesting a contribution to the risk of psychosis (57–59). For instance, family criticism has been associated with psychosis proneness, particularly in developing countries (60), and with the worsening of symptoms at the CHR stage (61). On the other hand, although less studied, positive characteristics of the family environment, such as family warmth, have been identified as protective factors (62–64).

The identification of sociodemographic and psychosocial factors that contribute to CHR states for psychosis across different populations and cultural contexts can broaden our understanding of risk and protective processes (65). Such research is crucial to inform the design of preventive strategies that can target modifiable factors (12, 66, 67) at a moment free of the confounding effects associated with clinical status, such as medication, chronicity, hospitalization, and social marginalization (68). Currently, research in this field is scarce in low- and middle-income countries (69). As such, further work is needed to identify factors associated with psychosis risk in different contexts than those of high-income countries (mainly European countries, Australia, and the United States), where most studies have been conducted with samples that do not represent the heterogeneity of the world’s population (70).

Mexico is one such country where scant research in this field has been conducted and where more regionally adapted knowledge is required to guide the development of specialized early psychosis services that are almost nonexistent (71). Furthermore, the Mexican population has experienced a range of adverse conditions, such as extreme poverty, social inequality, insecurity, and widespread violence. In addition, the population has been continuously impacted by natural disasters, such as earthquakes and floods. The above circumstances, taken together with the stress and disruptions associated with the COVID-19 pandemic, add further support to the relevance of examining sociodemographic and psychosocial factors associated with the risk for psychosis in this population. Therefore, this study used a Mexican general population sample to explore (i) sociodemographic and psychosocial differences between individuals with and without a positive screen for CHR, and (ii) sociodemographic and psychosocial factors associated with screening positive for CHR.

## 2. Materials and methods

### 2.1. Participants and procedure

The data were collected as part of an ongoing research project examining risk and protective factors for subclinical psychopathology in the Mexican general population. The current sample was recruited between March and July 2022 with individuals who voluntarily agreed to take part in an online survey administered *via* Qualtrics® software. For the present study, inclusion criteria were being born and currently residing in Mexico and being between 15 and 45 years old. Given the study’s focus on identifying predictors of CHR, exclusion criteria were self-reporting a psychotic spectrum disorder or a psychosis-related hospitalization.

The invitation to participate in the survey was distributed to the general population *via* personal and institutional (health and educational institutions) social media channels. All participants aged 18 and older provided informed consent. Youth were additionally invited to voluntarily participate through their high schools. School authorities sent information about the purposes of the study to parents or guardians *via* institutional e-mail and asked them to provide informed consent for their children’s participation. The survey was administered to minors authorized by their parents/guardians to participate and who provided their informed assent. Participants did not receive compensation for completing the survey. The study was approved by the Research Ethics Committee of the *Ramón de la Fuente Muñiz* National Institute of Psychiatry (CEI/C/019/2021) and conformed to the Helsinki Declaration.

A total of 866 individuals completed the survey. Of these, 38 were excluded for not meeting the inclusion criteria. We also excluded six participants who self-reported a psychotic disorder or a psychosis-related hospitalization. Thus, the analytic dataset included 822 participants.

### 2.2. Measures

#### 2.2.1. Sociodemographic questionnaire

Participants provided sociodemographic information, including age, sex, marital/relationship status, place of birth and residence, educational level (highest completed degree), current occupation, and socio-economic status based on monthly family income: low < 9,000 MXN; medium > 9,000 and < 45,000 MXN; high > 45,000 MXN (1 MXN is equivalent to approximately .05 USD). They also provided information about prior history of mental health difficulties and the type of difficulty experienced.

#### 2.2.2. CHR screening

The screening of CHR for psychosis status was based on two measures. The first was the 21-item Prodromal Questionnaire-Brief (PQ-B) (72, 73), a reliable and widely-used psychosis-risk screening instrument (74). Each PQ-B item is rated dichotomously (yes/no), and endorsed items are further rated on a 5-point distress scale. Following Fonseca-Pedrero et al. (72), we used the following PQ-B cut-off criteria: more than 6 positively endorsed items and a score equal to or above 29 on the distress scale. The second measure was the Social Functioning Questionnaire (SFQ) (75), which assesses functioning across diverse life domains, such as social contacts, work and home tasks, and leisure activities. Our use of this measure is in keeping with prior recommendations of considering low functioning as a criterion to identify CHR individuals (17, 76). The SFQ consists of 8 items rated on a 4-point scale, with higher scores indicating worse functioning. SFQ sum scores of 10 or higher are indicative of poor social functioning (75). Accordingly, participants were assigned to the CHR-positive group if they met the established cut-off scores on both the PQ-B and SFQ.

#### 2.2.3. Childhood maltreatment

The 28-item Childhood Trauma Questionnaire (CTQ) (77, 78) was used to assess sexual abuse, physical abuse, emotional abuse, emotional neglect, and physical neglect. CTQ items are answered on a 5-point scale ranging from “never true” to “very often true” and are summed to obtain a score for each type of maltreatment. Higher scores indicate greater levels of maltreatment. Consistent with current recommendations in the psychosis literature (79), we used the thresholds outlined by Walker et al. (80) to dichotomize each type of maltreatment according to the presence of clinically significant levels of exposure. These thresholds are as follows: a score of 15 or higher for emotional neglect, 10 or higher for emotional abuse, and 8 or higher for sexual abuse, physical abuse, and physical neglect.

#### 2.2.4. Other adverse experiences

Additional items were included to broaden the coverage of lifetime traumatic experiences. Specifically, we used 3 items adapted from the Brief Trauma Questionnaire (BTQ) (81) and 2 items adapted from the Questionnaire of Stressful Life Events (QSLE) (82). The items adapted from the BTQ assessed exposure to a major natural disaster, having lived in a place of war/terror or exposure to casualties, and experiencing the violent or unexpected death of a close family member or friend. The items adapted from the QSLE assessed exposure to peer bullying and intimate partner violence. Items were answered dichotomously (yes/no) and examined separately in the statistical analyses.

#### 2.2.5. COVID-related distress

The level of distress associated with the COVID-19 pandemic and lockdown was assessed using the following question: “*On a scale from 0 (not at all) to 10 (very much), how much distress has the pandemic and COVID-19 lockdown caused you?*,” with higher scores indicating greater levels of COVID-related distress.

#### 2.2.6. Cannabis risk level

The Alcohol, Smoking, and Substance Involvement Screening Test (ASSIST) (83, 84) was used to determine the level of risk associated with cannabis use. It comprises 8 items that assess the degree of substance involvement for several substances, including cannabis. Specifically, items measure lifetime and current (past 3 months) use, urge to consume, problems associated with use, failure to complete role obligations due to use, the concern expressed by others regarding consumption, and failure to stop or cut down use. The ASSIST provides a dimensional risk score for cannabis (a sum score that ranges from 0 to 39), which determines the level of risk: lower (0–3), moderate (4–26), or high (27 or more). Individuals with lower risk are not considered to require intervention, whereas those with moderate and high risk are deemed to require intervention. Previous research has supported the use of computer- and web-based modalities to administer the ASSIST (85, 86).

#### 2.2.7. Family functioning

The 6-item general functioning subscale (GF6+) (87) of the McMaster Family Assessment Device (FAD) (88, 89) was used to assess overall family functioning. Items are scored on a 4-point scale ranging from “strongly agree” to “strongly disagree,” with higher scores denoting worse family functioning. A mean score higher than 2 is indicative of an unhealthy family functioning.

### 2.3. Statistical analyses

For sample description, frequencies and percentages were used to summarize categorical variables, and means and standard deviations (S.D) were calculated for the continuous variables. For testing normality, skewness and kurtosis of the variables were calculated. A skewness value over 2 and kurtosis value over 7 are considered to be indicative of moderate or important non-normality (90). Overall, the skewness and kurtosis ranges found in our variables were not out of the norm, with a skewness range from −1.37 to 2.21 and a kurtosis range between −1.98 and 4.76. The only variable that exhibited a higher outside range for skewness (3.92) and kurtosis (13.41) was the item asking whether participants had lived in a place of war/terror or had been exposed to casualties. Given the limited evidence of non-normality, Chi-square tests (*χ*^2^) and independent sample Student’s *t*-tests were used to compare the study variables between those who screened positive for CHR (CHR-positive group) and those who did not (Non-CHR group). The sociodemographic (age, sex, marital/relationship status, educational level, current occupation, and socio-economic status) and psychosocial factors (abuse and neglect during childhood, traumatic events, the risk of presenting problems associated with the use of cannabis, and family functioning) were included as possible predictors associated with a CHR-positive screen in a multivariate logistic regression analysis using the backward conditional modeling approach. To determine the goodness of fit of the models, the Hosmer-Lemeshow test was used (91). Some variables were classified (dummy coded) into auxiliary variables to perform this analysis using their defined cut-off points and were represented by two values, “0” and “1,” where the latter represents the risk/present value. In particular, the variables were coded as follows: marital/relationship status (0 = partnered, 1 = divorced/separated, single), socio-economic level (0 = medium and high, 1 = low), educational level (0 = bachelor studies and higher, 1 = high school studies or less), current occupation (0 = student, economically remunerated activity, 1 = unemployed, non-remunerated activity), cannabis risk level (0 = low risk, 1 = moderate/high risk), family functioning (0 = healthy functioning, i.e., score ≤ 2, 1 = unhealthy functioning, i.e., score > 2), and the previously described cut-offs for clinically significant exposure for the CTQ maltreatment subscales. Two models are presented, the first one containing all the variables in the first step of the modeling approach, and the second including only those variables that remained in the model after the backward stepwise process and which has the most adequate goodness of fit. All tests were deemed to be significant with a *p* ≤ 0.05. For the analyses, the SPSS version 21 statistical package was used.

## 3. Results

### 3.1. Sociodemographic factors

The final study sample consisted of 822 individuals. The sample was predominantly female (78.2%, *n* = 643), with a mean age of 29.1 years (S.D. = 8.3, range 15–45). Most participants were single (55.5%, *n* = 456; with partner = 40.6%, *n* = 334; separated/divorced 3.9%, *n* = 32), with a higher percentage reporting at least a bachelor’s degree (64.4%, *n* = 529) as well as having an economically remunerated activity (51.9%, *n* = 427) or being students (35.8%, *n* = 294). Less than half of the sample self-reported a mental health problem (32.8%, *n* = 270). Of those with a reported mental health problem, affective (28.1%, *n* = 76) and anxious symptomatology (27.8%, *n* = 75) were the most frequently reported. The remaining sociodemographic features are displayed in Table 1.

**Table 1:** Comparison of sociodemographic factors between the CHR-positive and Non-CHR groups.

	Total *n* = 822	Non-CHR *n* = 680	CHR-positive *n* = 142	Statistics
**Demographic features *n* %**				
**Sex**–Women	643 78.2	536 78.8	107 75.4	χ^2^ = 0.8, *p* = 0.36
**Age**–years (mean; S.D.)	29.1 8.3	29.7 8.3	26.4 7.6	*t* = 4.2, *p* < 0.001
**Marital/relationship status**				
Single	456 55.5	373 54.9	83 58.5	χ^2^ = 5.3, *p* = 0.14
Married/cohabiting	185 22.5	163 24.0	22 15.5	
Has a romantic partner	149 18.1	118 17.4	31 21.8	
Separated/divorced	32 3.9	26 3.8	6 4.2	
**Level of completed education**				
Elementary school	2 0.2	2 0.3	0	χ^2^ = 29.7, *p* < 0.001
Secondary school	24 2.9	15 2.2	9 6.3	
Technical career	11 1.3	8 1.2	3 2.1	
High school	256 31.1	193 28.4	63 44.4	
Bachelor studies	370 45.0	315 46.3	55 38.7	
Postgraduate	159 19.3	147 21.6	12 8.5	
**Current occupation**				
Student/economically remunerated activity	721 87.7	603 88.7	118 83.1	χ^2^ = 3.3, *p* = 0.06
Unemployed/non-remunerated activity	101 12.3	77 11.3	24 16.9	
Socioeconomic status*				
Low	262 31.9	207 30.4	55 38.7	χ^2^ = 5.8, *p* = 0.053
Medium	502 61.1	420 61.8	82 57.7	
High	58 7.1	53 7.8	5 3.5	
**Self-reported mental health problem**–Yes	270 32.8	198 29.1	72 50.7	χ^2^ = 24.8, *p* < 0.001

*Low: < 9,000 MXN; medium: ≥ 9,000 and ≤ 45,000 MXN; high: > 45,000 MXN.

When dividing the sample into the CHR-positive group and the Non-CHR group, 17.3% (*n* = 142) met the combined PQ-B and SFQ criteria for a CHR-positive screen. As reported in Table 1, no differences emerged between the groups in terms of sex, marital/relationship status, current occupation, or socio-economic status. Nevertheless, a higher percentage of individuals from the CHR-positive group reported lower educational levels, more self-reported mental health problems, and were younger than those in the Non-CHR group.

### 3.2. Psychosocial factors

Most of the participants reported a low risk of presenting problems associated with cannabis consumption (87.1%, *n* = 716). However, a higher percentage of CHR-positive individuals reported medium/high risk compared to the Non-CHR group. As displayed in Table 2, in terms of experiences of adversity, no differences arose between groups regarding the experience of living in a place of war/terror (5.5%, *n* = 45 of the total sample) or experiencing a major natural disaster (20.2%, *n* = 166 of the total sample). Nevertheless, a higher percentage of those screening positive for CHR experienced a violent or unexpected death of a relative or friend, peer bullying, and intimate partner violence. This group also reported poorer family functioning, more distress associated with the COVID-19 pandemic and lockdown measures, and higher levels of childhood maltreatment across all the CTQ subscales. The differences in childhood maltreatment also emerged when considering the thresholds for the presence of clinically significant levels of exposure. In particular, as compared with the Non-CHR group, the CHR-positive group reported higher rates of emotional abuse (81.7%, *n* = 116 vs. 48.5%, *n* = 330; *p* < 0.001), physical abuse (62.0%, *n* = 88 vs. 36.6%, *n* = 249; *p* < 0.001), sexual abuse (43.7%, *n* = 62 vs. 25.7%, *n* = 175; *p* < 0.001), emotional neglect (49.3%, *n* = 70 vs. 22.8%, *n* = 155; *p* < 0.001), and physical neglect (57.7%, *n* = 82 vs. 31.2%, *n* = 212; *p* < 0.001).

**Table 2:** Comparison of psychosocial factors between the CHR-positive and Non-CHR groups.

	Total *n* = 822	Non-CHR *n* = 680	CHR-positive *n* = 142	Statistics
**Cannabis risk level *n* %**				
Cannabis risk level–moderate/high	106 12.9	68 10.	38 26.8	χ^2^ = 29.3, *p* < 0.001
**Adverse experiences *n* %**				
Lived in a place of war/terror–yes	45 5.5	38 5.6	7 4.9	χ^2^ = 0.09, *p* = 0.75
Experienced major natural disaster–yes	166 20.2	129 19.0	37 26.1	χ^2^ = 3.6, *p* = 0.056
Experienced a violent or unexpected death–yes	319 38.8	248 36.5	71 50.0	χ^2^ = 9.0, *p* = 0.003
Experienced bullying–yes	438 53.3	338 49.7	100 70.4	χ^2^ = 20.2, *p* < 0.001
Experienced intimate partner violence–yes	406 49.4	324 47.6	82 57.7	χ^2^ = 4.7, *p* = 0.02
**Childhood maltreatment Mean S.D.**				
Emotional abuse	11.2 5.4	10.4 5.0	15.1 5.5	*t* = −9.9, *p* < 0.001
Physical abuse	8.2 4.2	7.8 3.8	10.2 5.1	*t* = −5.3, *p* < 0.001
Sexual abuse	7.8 4.7	7.5 4.5	8.9 5.3	*t* = −2.8, *p* < 0.001
Emotional neglect	11.4 4.8	10.9 4.7	14.0 4.8	*t* = −7.0, *p* < 0.001
Physical neglect	7.4 3.0	7.1 2.9	8.9 3.3	*t* = −5.7, *p* < 0.001
Total score	46.2 16.9	43.9 15.6	57.2 18.6	*t* = −7.9, *p* < 0.001
**Family functioning**				
Family functioning (mean S.D.)	2.0 0.7	1.9 0.7	2.5 0.6	*t* = −8.7, *p* < 0.001
Unhealthy family functioning–yes (*n* %)	383 46.6	273 40.1	110 77.5	χ^2^ = 65.7, *p* < 0.001
**COVID-related distress Mean S.D.**				
COVID-related distress	6.08 2.52	5.9 2.5	6.9 2.5	*t* = −4.2, *p* < 0.001

### 3.3. Factors associated with screening positive for a CHR for psychosis

The variables associated with screening positive for CHR for psychosis in the present sample were: having an unhealthy family functioning, a higher risk related to cannabis use, a lower level of completed education, having experienced a major natural disaster, having experienced a violent or unexpected death of a relative or friend, clinically significant levels of emotional abuse, physical neglect, and physical abuse during childhood, and higher distress associated with the COVID-19 pandemic. An older age was a protective factor for screening positive for CHR. The results of the initial and final logistic regression models are displayed in Table 3.

**Table 3:** Factors associated with screening positive for CHR for psychosis.

	Initial Model			Final Model		
	OR	95% C.I.		OR	95% C.I.	
Sex–men	1.34	0.79	2.25	-	-	-
Age	0.95**	0.91	0.98	0.96*	0.92	0.99
Marital/relationship status–single/not partnered	0.81	0.52	1.28	-	-	-
Level of completed education–high school or less	1.52	0.92	2.50	1.55*	1.003	2.54
Current occupation–unemployed/not remunerated	1.40	0.77	2.53	-	-	-
Socioeconomic status–low	1.11	0.71	1.73	-	-	-
Cannabis risk level–moderate/high	2.63***	1.53	4.49	2.75***	1.63	4.64
Lived in a place of war/terror–yes	0.89	0.34	2.31	-	-	-
Experienced major natural disaster–yes	1.90*	1.15	3.15	1.94**	1.18	3.16
Experienced a violent or unexpected death–yes	1.83**	1.20	2.79	1.85**	1.22	2.81
Experienced bullying–yes	1.43	0.90	2.28	-	-	-
Experienced intimate partner violence–yes	1.12	0.71	1.78	-	-	-
Emotional abuse–clinically significant	1.73*	1.002	3.07	1.88*	1.09	3.25
Physical abuse–clinically significant	1.53	0.96	2.43	1.66*	1.05	2.61
Sexual abuse–clinically significant	1.34	0.84	2.14	-	-	-
Emotional neglect–clinically significant	1.23	0.74	2.03	-	-	-
Physical neglect–clinically significant	1.50	0.93	2.40	1.68*	1.08	2.61
Family functioning–unhealthy	2.58***	1.56	4.25	2.75***	1.69	4.46
COVID-related distress	1.11	1.02	1.21	1.10*	1.01	1.20
Initial model: *R*^2^ Nagelkerke = 0.31 Test Hosmer and Lemeshow = *p* = 0.34 Final model: *R*^2^ Nagelkerke = 0.30 Test Hosmer and Lemeshow = *p* = 0.14						

*p ≤ 0.05, **p ≤ 0.01***p ≤ 0.001 Hyphen (−) indicates variables which did not enter in the regression model.

## 4. Discussion

To the best of our knowledge, this is the first study to explore sociodemographic and psychosocial factors associated with the risk of developing psychosis in a Mexican sample. Our results showed that the CHR-positive group had higher levels of environmental risk than the Non-CHR group. Furthermore, regarding the variables associated with a CHR-positive screen, an unhealthy family functioning and cannabis risk level were the strongest predictors. In addition, consistent with current evidence in the literature, age, educational level, childhood maltreatment, and traumatic events were also associated with screening positive for CHR. Finally, in line with emerging work on the mental health impact of the COVID-19 pandemic, COVID-related distress also emerged as a significant risk factor. Overall, these results add to current efforts to identify commonalities and specificities of risk factors for psychosis across different sociocultural contexts.

We found that 17.3% (*n* = 142) of this Mexican sample screened positive for a CHR for psychosis, which is higher than the rate (1.7%) reported in the most recent systematic review and meta-analysis of the prevalence of CHR in the general population (18). There are several potential reasons for this finding. First, this could be associated with the evidence that the prevalence rates of psychotic-like experiences vary across countries of different income levels, with a higher prevalence reported in low- and middle-income countries than in high-income countries (7, 70). Second, the prevalence in this sample could be related to the recent impact of the COVID-19 pandemic. Worldwide, several studies have shown an increase in mental health problems in the general population during the COVID-19 pandemic (92–95), including an increase in psychotic-like experiences (9, 10, 11, 96). However, due to the lack of pre-pandemic studies on the prevalence of CHR in the Mexican population, we are unable to draw any conclusions regarding the impact of the pandemic in this respect. Additionally, the higher prevalence could also be due to the use of self-report measures for the designation of risk status. Future studies using interview measures for the detection of CHR individuals in the Mexican general population may help clarify the potential impact of the assessment method on prevalence rates.

### 4.1. Differences between CHR-positive and non-CHR groups

The findings of the comparisons between the CHR-positive and Non-CHR groups showed that individuals screening positive for CHR were younger and reported lower educational levels than those in the Non-CHR group. These differences might be interrelated, as the lower level of highest education attained by the CHR-positive group may correspond to their average younger age. Moreover, the CHR-positive group self-reported more mental health problems than the Non-CHR group, which is consistent with the clinical features of the at-risk stages described in the literature, characterized by the presence of a wide range of non-specific psychopathology and a high prevalence of non-psychotic psychiatric comorbidity, especially with depressive and anxiety disorders (97–100).

The findings regarding differences in the psychosocial domain are in line with previous studies indicating that cannabis misuse, childhood maltreatment, bullying, and traumatic events are more prevalent among CHR individuals, as compared with healthy controls or the general population (32, 37, 47, 67, 101–103). We also found that the CHR-positive group reported poorer family functioning and more distress associated with the COVID-19 pandemic than the Non-CHR group. Although comparisons on these variables are scarcer in the literature, the findings are consistent with the evidence that CHR individuals report worse family functioning or less family satisfaction than Non-CHR individuals (104–106). In addition, they support the results of a recent study suggesting an association between psychotic-like experiences and a greater psychological impact of the COVID-19 pandemic (107). Further research on the effects of the COVID-19 pandemic on this population is needed to confirm this finding and its potential clinical implications.

### 4.2. Factors associated with a positive screen for CHR

In line with prior studies (e.g., 10, 40), findings regarding sociodemographic characteristics showed that being older is a protective factor, as it was associated with a lower risk of screening positive for CHR. Furthermore, a lower educational level emerged as a risk factor. Although this supports what has been shown in previous work (10, 44), we interpret this finding with caution, as the most frequent level of education completed by the CHR-positive individuals in our sample was high school, which represents a high or above-average educational level for the Mexican population (108). Therefore, more research in larger and more representative Mexican samples is required to confirm it as a risk factor.

This study found that an unhealthy family functioning was an important psychosocial factor associated with screening positive for CHR for psychosis. This result is notable in light of the centrality of family relationships in the Mexican sociocultural context (109, 110) and seems consistent with the finding that the sociocultural context influences the extent to which components of family functioning are linked to psychotic-like experiences, with a stronger association in developing countries than in highly industrialized countries (60). Future studies directly assessing cultural processes in the Mexican context can enrich our understanding of how culture shapes the interrelations between family factors and psychosis (65, 111, 112). Although family functioning has been less extensively investigated as a predictor of CHR states, existing research on the family environment has indicated that negative family affect and family stress (e.g., criticism, communication problems, less support and cohesion) are associated with poor outcomes across the psychosis continuum, including young people vulnerable to psychosis (55, 56, 58, 113–115), while certain components of a positive family environment (e.g., warmth, positive remarks, family support, cohesion and adaptability, quality of parent–child communication) seem to act as protective factors in the early course of psychosis and have been found to predict improved symptoms or functioning over time in CHR individuals (54, 105, 116–118). Since psychotic symptoms generally emerge during adolescence and early adulthood, a period when individuals are typically greatly impacted by family interactions, these findings suggest the relevance of tailoring early family interventions to bolster family functioning and improve family communication, such as by emphasizing supportive interactions and family psychoeducation (117–120).

Our findings also showed that having a moderate/high risk related to cannabis use was associated with a CHR-positive screen, which is in line with previous research demonstrating an association of cannabis use with psychotic symptoms and psychotic disorders (121–123). Although there is strong evidence indicating a dose–response relationship between cannabis use and psychosis (27, 124, 125), conflicting findings have also emerged (19) and the evidence of the effect of cannabis use on the exacerbation of attenuated psychotic symptoms in CHR individuals is inconsistent (33, 126). Current research on cannabis and psychosis has highlighted several moderating factors, including genetic variability as well as the frequency and age of onset of cannabis use (127, 128). Thus, it will be essential for future research conducted in the Mexican context to examine these factors, especially considering that the COVID-19 pandemic may have impacted the patterns and frequency of cannabis use (129, 130) and that the mean age of onset of cannabis use in Mexico has been decreasing in recent years (131).

Consistent with a robust body of work, we found that early and subsequent exposure to psychosocial stress and adversity was associated with vulnerability to psychosis (12, 27, 30, 37, 132, 133). In particular, findings showed that a CHR-positive screen was associated with having experienced higher levels of emotional abuse, physical abuse, and physical neglect during childhood, a major natural disaster, violent or unexpected death of a relative or friend, and higher distress due to the COVID-19 pandemic.

The association of emotional abuse, physical abuse, and physical neglect with screening positive for CHR parallels previous research demonstrating that a history of childhood maltreatment represents a strong risk factor for psychotic phenomena and the manifestation of the CHR state (19, 37, 39, 42, 47, 134). Furthermore, the findings regarding emotional abuse are in line with a recent review showing that emotional abuse is the type of adversity most strongly associated with subclinical expressions of psychosis in non-clinical samples (135). Therefore, our results concerning childhood maltreatment support those obtained across other populations and further highlight the relevance of distinguishing among maltreatment types when investigating associations with psychosis risk.

Interestingly, some of the other environmental experiences associated with screening positive for CHR in this sample, such as having experienced a major natural disaster and the violent or unexpected death of a relative or friend, have been less prominent in the literature but seem consistent with the particular adverse life experiences of the Mexican population. In recent years, the violence in Mexico has increased substantially, with a historical record of femicides, homicides, and forced disappearances due to organized crime (136). Furthermore, over recent decades, the Mexican population has experienced multiple natural disasters, such as the 2017 earthquakes, as well as floods caused by hurricanes and tropical storms that have resulted in numerous deaths and many victims (*damnificados*). In addition, although studies are still limited, the daily stressors during the COVID-19 pandemic have impacted the mental health of the Mexican population (137)—and our results suggest that this stress may also be associated with the risk of psychosis. Therefore, further epidemiological and longitudinal research with larger samples is required to better understand the potential medium- and long-term impact of the psychosocial stress and traumatic events identified as risk factors in the present study.

### 4.3. Clinical implications

The findings of this study have several clinical implications. The high rate of individuals with a CHR-positive screen in this Mexican sample is alarming and underscores the need to prioritize and promote early detection programs and preventive interventions focused on the early stages of psychosis, which are almost nonexistent in Mexico. Furthermore, if replicated, the psychosocial variables identified as risk factors could help to guide preventive interventions in the population with potential risk for developing psychotic disorders. For instance, both family functioning and cannabis use are amenable to intervention. Therefore, preventive strategies and early interventions could benefit from a focus on reducing dysfunctional family dynamics (54), as well as educational, psychological, and social interventions to reduce cannabis use and its associated risks in CHR adolescents and young adults (31). In addition, the findings support prior calls to make routine and sensitive inquiries about childhood adversity when working with individuals presenting with psychosis-spectrum psychopathology, as well as the importance of implementing effective treatments for childhood trauma in this population (67, 138, 139). Moreover, considering the context of humanitarian emergencies and generalized violence that the Mexican population has experienced, it is necessary to create crisis-focused interventions to reduce the impact of these events on people’s mental health and psychosocial well-being (140).

### 4.4. Limitations

The current research has certain limitations that should be considered when interpreting the results. First, the cross-sectional nature of the study limits the inferences that can be drawn in terms of causality. Second, all measures were self-reports, which are susceptible to reporting biases. Concerning the designation of a CHR status through self-report measures, we note that we attempted to mitigate this limitation by using strict criteria that included both PQ-B and SFQ cut-offs to establish a CHR-positive screen and that previous work has supported the possibility of detecting CHR individuals through online screening (141). However, it would have been preferable to use widely-validated semi-structured interviews, such as the SIPS (16) or the CAARMS (13). In this regard, it is relevant to note that a recent large-scale study found that both self-report and interview measures of psychotic experiences were associated with similar risk indicators (142). Finally, our use of a non-random sampling technique (participants were individuals with Internet access who were interested in responding to the survey) and the fact that the sample was predominantly female with a relatively high educational level limit the generalizability of the results.

### 4.5. Conclusion

In closing, this study contributes to the body of research examining sociodemographic and psychosocial factors associated with an elevated risk for psychosis across different countries and populations. In particular, we provided, to our knowledge, the first examination of this topic in the Mexican population. Several of the risk factors that emerged as relevant in the present study have been widely reported in the literature, while the significance of other, less extensively studied factors seems consistent with the sociocultural fabric and experiences of the Mexican population. The current work represents a starting place for future studies to build upon, especially by examining these factors longitudinally as well as their interactions with genetic susceptibility. Overall, this study highlights the importance of examining potential contributors to psychosis vulnerability across different sociocultural contexts to delineate risk and protective processes relevant to specific populations and better target preventive intervention efforts.

## Data availability statement

The datasets presented in this article are not readily available due to ethical restrictions regarding the privacy of research participants. Requests to access the datasets should be directed to tecellid@imp.edu.mx.

## Ethics statement

The studies involving human participants were reviewed and approved by the Research Ethics Committee of the Ramón de la Fuente Muñiz National Institute of Psychiatry (CEI/C/019/2021). All participants aged 18 and older provided informed consent. Minor participants provided informed assent and their parents/legal guardians provided informed consent.

## Author contributions

TD-M was the principal investigator, oversaw data collection, conceived the study, and contributed to writing—original draft, review, and editing. TS contributed to the scoring/management of the data and writing—original draft, review, and editing. AF conducted the data analysis and contributed to writing—original draft, review, and editing. All authors critically revised the manuscript for important intellectual content and approved the submitted version.

## Funding

This study was supported by Programas Nacionales Estratégicos del Consejo Nacional de Ciencia y Tecnología (CONACyT-PRONACES), grant 3205.

## Conflict of interest

The authors declare that the research was conducted in the absence of any commercial or financial relationships that could be construed as a potential conflict of interest.

## Publisher’s note

All claims expressed in this article are solely those of the authors and do not necessarily represent those of their affiliated organizations, or those of the publisher, the editors and the reviewers. Any product that may be evaluated in this article, or claim that may be made by its manufacturer, is not guaranteed or endorsed by the publisher.
